# Supplementary material for: Bat dynamics modelling as a tool for conservation management in subterranean environments
Source: PLoS One. 2022 Oct 20;17(10):e0275984. doi: 10.1371/journal.pone.0275984 (PMC9584375; doi:10.1371/journal.pone.0275984)
Supplement: S1 File — (DOCX) [file pone.0275984.s001.docx]

**Supplementary materials**

*
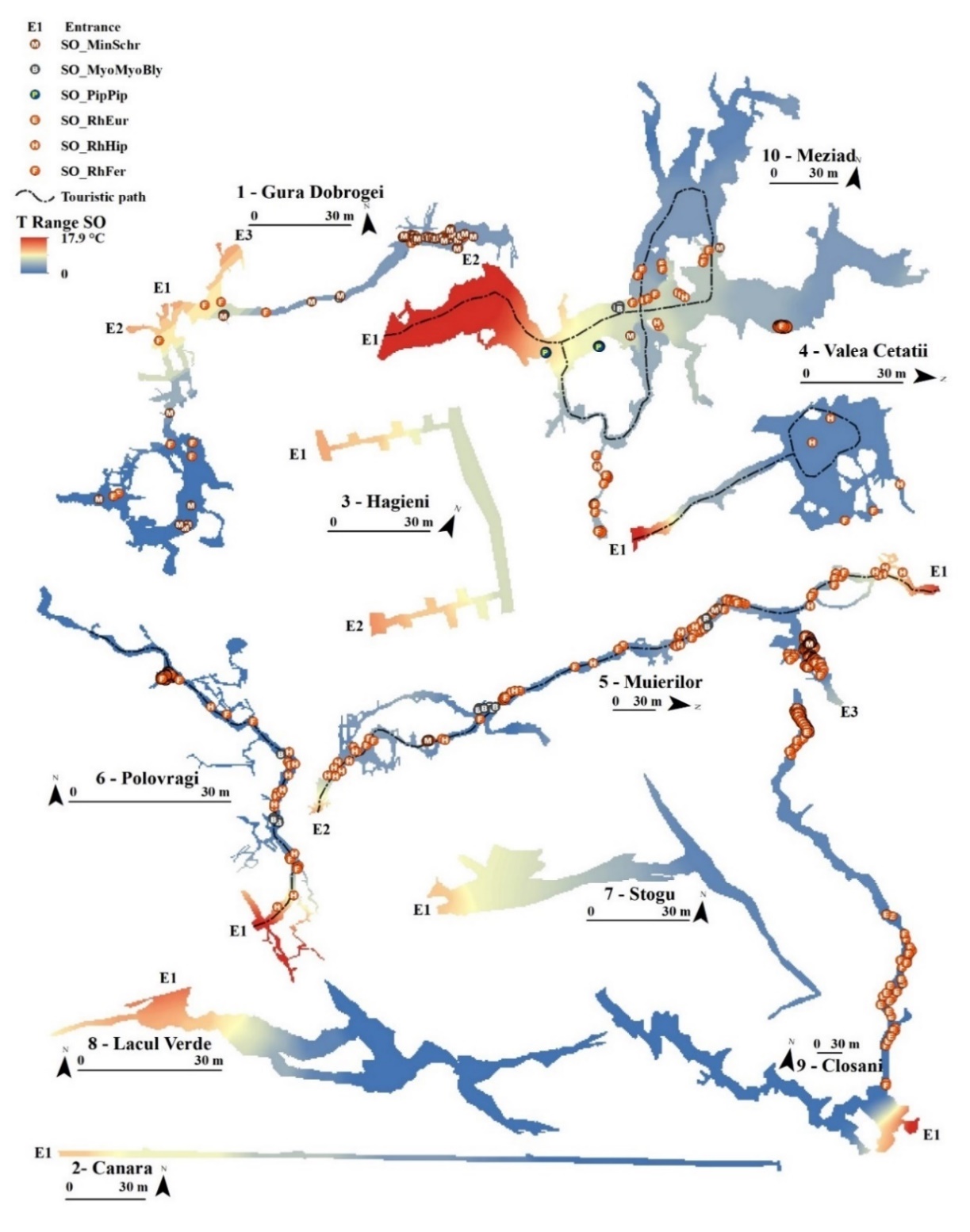
*

Figure S1 – Bat distribution and temperature range during SO

*MinSchr – Miniopterus schreibersii, MyoMyoBly – Myotis myotis/blythii, PipPip – Pipistrellus pipistrellus RhEur – Rhinolophus euryale, RhHip – Rhinolophus hipposideros, RhFer – Rhinolophus ferrumequinum.*

*
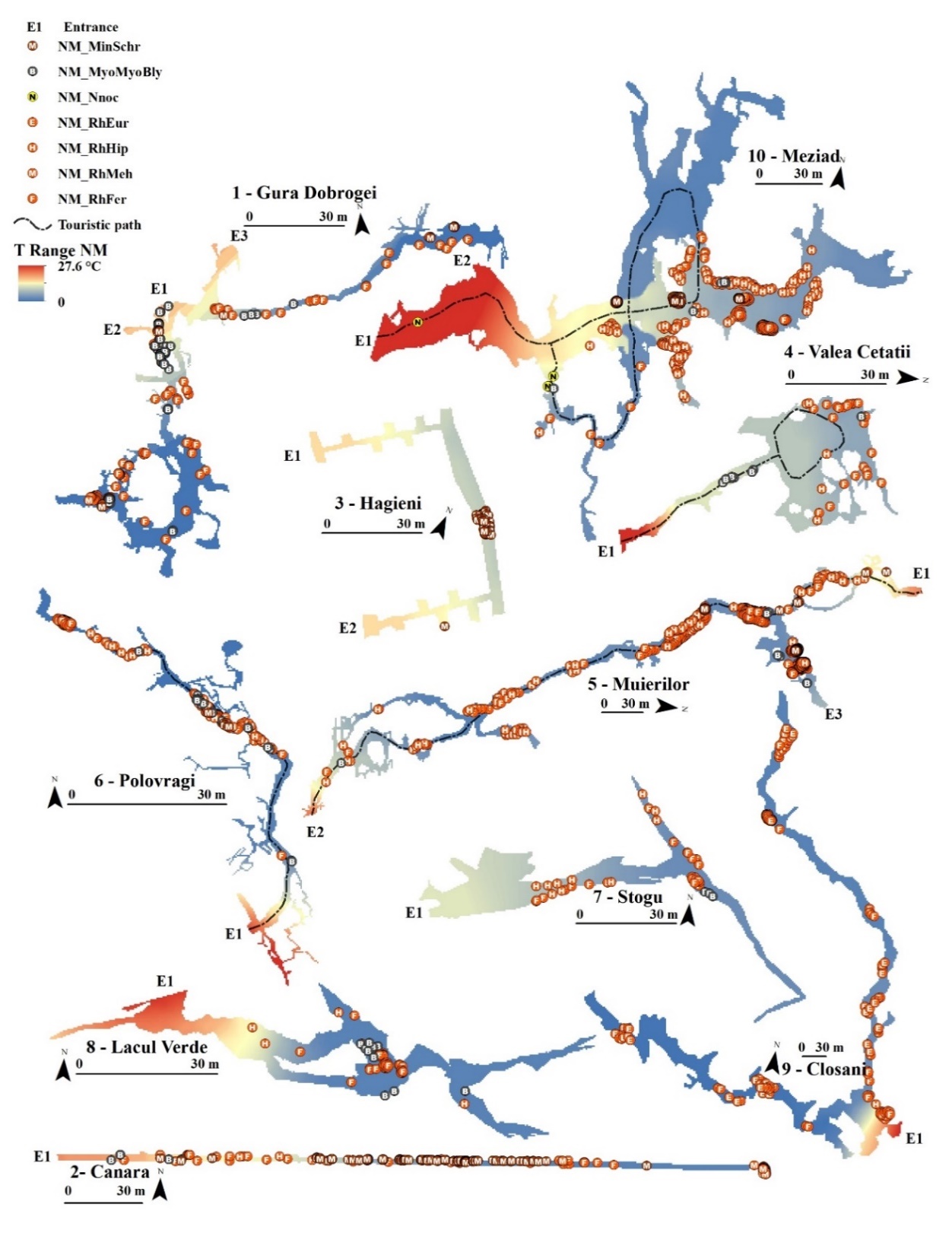
*

Figure S2 – Bat distribution and temperature range during NM

*MinSchr – Miniopterus schreibersii, MyoMyoBly – Myotis myotis/blythii, Nnoc – Nyctalus noctula, RhEur – Rhinolophus Euryale, RhHip – Rhinolophus hipposideros, RhMeh – Rhinolophus mehelyi, RhFer – Rhinolophus ferrumequinum.*

**
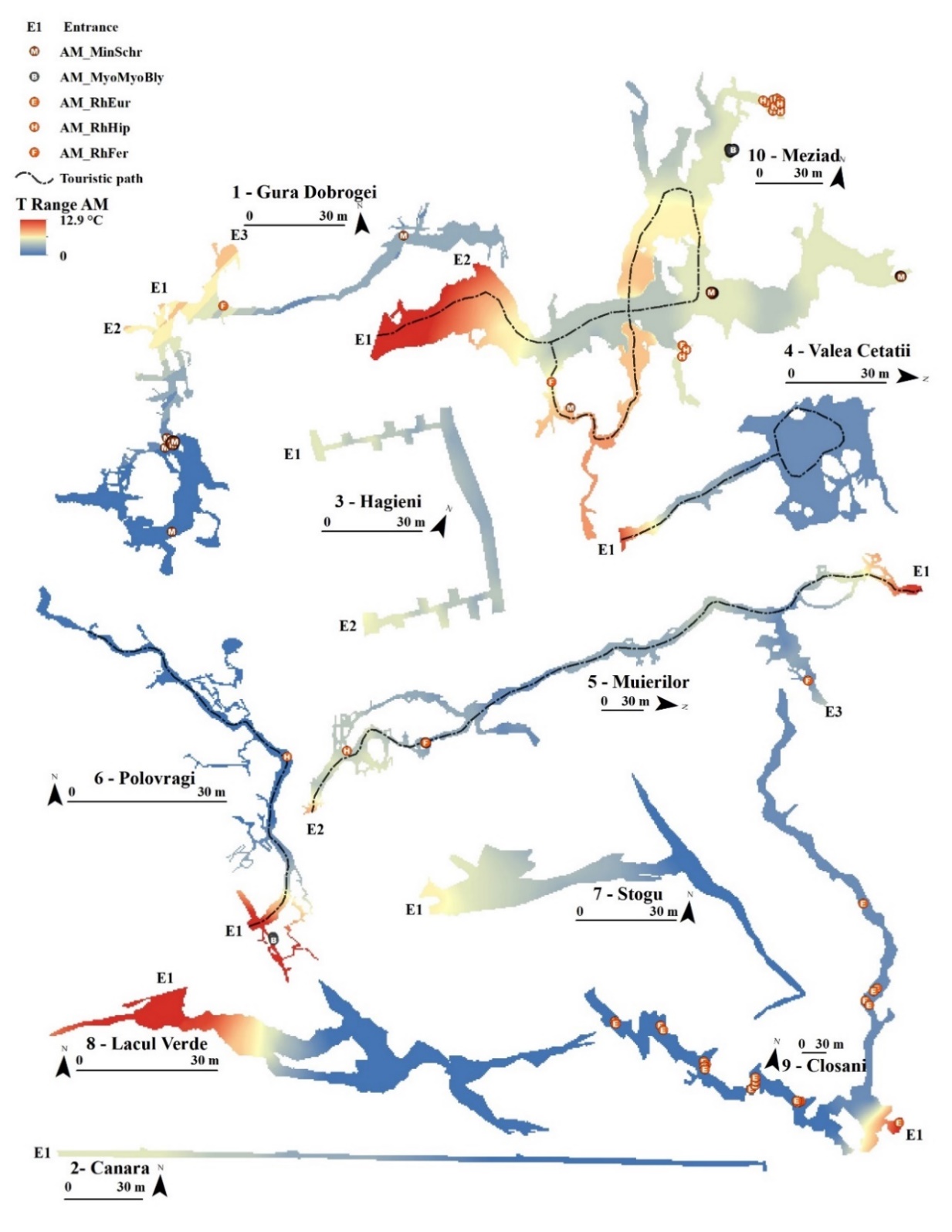
**

Figure S3 – Bat distribution and temperature range during AM

*MinSchr – Miniopterus schreibersii, MyoMyoBly – Myotis myotis/blythii, RhEur – Rhinolophus euryale, RhHip – Rhinolophus hipposideros, RhFer – Rhinolophus ferrumequinum.*

Table S1 - Data logger average values for each observation period. Location of data loggers in Figure 2.

| No. | Cave | Logger code | November - March (NM) | | | | | September - October (SO) | | | | | April - May (AM) | | | | |
| --- | --- | --- | --- | --- | --- | --- | --- | --- | --- | --- | --- | --- | --- | --- | --- | --- | --- |
|  |  |  | Tmax NM | Tmean NM | Tmin NM | Trange NM | Tstd NM | Tmax SO | Tmean SO | Tmin SO | Trange SO | Tstd SO | Tmax AM | Tmean AM | Tmin AM | Trange AM | Tstd AM |
| **1** | Canara Tunnel | CA 1 | 15.49 | 9.01 | 0.99 | 14.51 | 2.56 | 17.56 | 13.98 | 9.81 | 8.00 | 1.83 | 19.67 | 17.70 | 9.61 | 4.00 | 1.08 |
| **2** | Canara Tunnel | CA 2 | 14.28 | 10.98 | 6.92 | 7.83 | 1.30 | 15.43 | 13.55 | 11.42 | 4.00 | 0.96 | 16.50 | 15.50 | 11.28 | 2.00 | 0.55 |
| **3** | Canara Tunnel | CA 3 | 13.10 | 13.02 | 12.86 | 1.00 | 0.05 | 13.21 | 13.11 | 13.01 | 0.00 | 0.08 | 13.21 | 13.20 | 13.03 | 0.00 | 0.00 |
| **4** | Cloşani cave | CL 1 | 15.91 | 5.68 | -4.25 | 19.25 | 3.73 | 22.03 | 13.21 | 7.11 | 14.84 | 3.58 | 22.74 | 18.11 | 5.46 | 9.61 | 2.01 |
| **5** | Cloşani cave | CL 2 | 11.25 | 10.12 | 9.06 | 2.00 | 0.56 | 11.60 | 11.39 | 11.14 | 0.00 | 0.12 | 11.41 | 10.93 | 9.78 | 1.00 | 0.26 |
| **6** | Cloşani cave | CL 3 | 11.31 | 10.99 | 10.74 | 0.59 | 0.15 | 11.38 | 11.26 | 11.14 | 0.00 | 0.09 | 11.30 | 11.15 | 10.84 | 0.39 | 0.10 |
| **7** | Cloşani cave | CL 4 | 11.30 | 11.16 | 11.05 | 0.00 | 0.07 | 11.31 | 11.22 | 11.14 | 0.00 | 0.08 | 11.23 | 11.16 | 11.05 | 0.00 | 0.07 |
| **8** | Cloşani cave | CL 5 | 11.26 | 11.17 | 11.10 | 0.00 | 0.03 | 11.22 | 11.18 | 11.14 | 0.00 | 0.04 | 11.19 | 11.15 | 11.10 | 0.00 | 0.04 |
| **9** | Cloşani cave | CL 6 | 11.29 | 11.17 | 11.12 | 0.00 | 0.03 | 11.18 | 11.16 | 11.14 | 0.00 | 0.02 | 11.16 | 11.14 | 11.12 | 0.00 | 0.02 |
| **10** | Cloşani cave | CL 7 | 11.21 | 10.44 | 9.66 | 2.00 | 0.39 | 11.41 | 11.29 | 11.14 | 0.00 | 0.07 | 11.32 | 11.00 | 10.18 | 1.00 | 0.17 |
| **11** | Cloşani cave | CL 8 | 11.27 | 10.77 | 10.28 | 1.00 | 0.22 | 11.34 | 11.17 | 11.06 | 0.33 | 0.07 | 11.33 | 11.08 | 10.60 | 1.00 | 0.11 |
| **12** | Cloşani cave | CL 9 | 11.34 | 11.11 | 10.92 | 1.00 | 0.13 | 11.34 | 11.06 | 10.94 | 1.00 | 0.13 | 11.34 | 11.15 | 10.98 | 1.00 | 0.11 |
| **13** | Gura Dobrogei cave | GD 1 | 13.65 | 7.83 | 0.58 | 13.00 | 2.19 | 17.08 | 13.15 | 7.61 | 10.00 | 2.18 | 19.20 | 16.66 | 8.07 | 6.00 | 1.20 |
| **14** | Gura Dobrogei cave | GD 2 | 11.60 | 11.48 | 11.40 | 0.00 | 0.06 | 11.80 | 11.68 | 11.50 | 0.00 | 0.08 | 11.80 | 11.75 | 11.60 | 0.00 | 0.05 |
| **15** | Gura Dobrogei cave | GD 3 | 10.98 | 10.50 | 10.20 | 0.00 | 0.30 | 12.00 | 11.67 | 10.98 | 2.00 | 0.31 | 12.00 | 11.75 | 10.40 | 2.00 | 0.35 |
| **16** | Hagieni Tunnel | HG 1 | 11.55 | 6.16 | -0.33 | 11.00 | 2.17 | 16.47 | 13.32 | 7.04 | 9.00 | 2.23 | 16.90 | 15.47 | 8.05 | 4.00 | 1.03 |
| **17** | Hagieni Tunnel | HG 2 | 10.89 | 6.53 | 3.89 | 6.81 | 1.62 | 13.54 | 11.91 | 8.52 | 5.00 | 1.16 | 13.08 | 12.39 | 7.58 | 2.81 | 0.58 |
| **18** | Hagieni Tunnel | HG 3 | 11.13 | 6.39 | 2.37 | 9.00 | 1.77 | 14.49 | 12.44 | 8.00 | 6.91 | 1.54 | 14.36 | 13.55 | 7.76 | 3.00 | 0.72 |
| **19** | Lacul Verde cave | LV 1 | 12.10 | 4.49 | -7.27 | 18.38 | 3.99 | 14.47 | 9.40 | 3.92 | 10.53 | 2.29 | 21.57 | 16.59 | 7.92 | 11.85 | 2.54 |
| **20** | Lacul Verde cave | LV 2 | 9.10 | 8.99 | 8.90 | 1.00 | 0.08 | 9.10 | 9.10 | 9.10 | 0.00 | 0.00 | 9.10 | 9.10 | 9.10 | 0.00 | 0.00 |
| **21** | Lacul Verde cave | LV 3 | 9.10 | 8.99 | 8.90 | 1.00 | 0.08 | 9.10 | 9.10 | 9.10 | 0.00 | 0.00 | 9.10 | 9.10 | 9.10 | 0.00 | 0.00 |
| **22** | Muierilor cave | MU 1 | 11.57 | 5.03 | -4.26 | 14.85 | 2.81 | 18.49 | 11.66 | 6.09 | 12.32 | 3.23 | 20.52 | 15.99 | 4.99 | 9.39 | 1.95 |
| **23** | Muierilor cave | MU 2 | 9.69 | 8.60 | 7.73 | 2.02 | 0.52 | 10.51 | 9.98 | 9.49 | 1.00 | 0.28 | 10.38 | 9.70 | 7.79 | 1.98 | 0.31 |
| **24** | Muierilor cave | MU 3 | 9.30 | 8.33 | 7.46 | 2.00 | 0.45 | 10.17 | 9.64 | 9.09 | 1.41 | 0.25 | 10.23 | 9.31 | 7.56 | 2.00 | 0.50 |
| **25** | Muierilor cave | MU 4 | 9.62 | 9.10 | 8.55 | 1.00 | 0.25 | 10.01 | 9.78 | 9.56 | 0.76 | 0.09 | 10.08 | 9.48 | 8.58 | 1.99 | 0.33 |
| **26** | Muierilor cave | MU 5 | 11.80 | 10.85 | 9.57 | 2.02 | 0.91 | 11.79 | 11.27 | 10.66 | 1.02 | 0.28 | 11.48 | 10.88 | 9.70 | 1.16 | 0.59 |
| **27** | Muierilor cave | MU 6 | 12.62 | 9.94 | 6.56 | 6.52 | 1.60 | 13.25 | 12.23 | 11.31 | 2.00 | 0.45 | 13.43 | 11.78 | 8.14 | 3.52 | 1.03 |
| **28** | Muierilor cave | MU 7 | 10.93 | 9.89 | 8.98 | 1.32 | 0.59 | 11.78 | 10.60 | 9.58 | 2.00 | 0.94 | 11.96 | 10.46 | 9.10 | 2.16 | 1.09 |
| **29** | Muierilor cave | MU 8 | 9.32 | 5.70 | -0.66 | 9.01 | 1.85 | 12.12 | 10.17 | 7.74 | 4.94 | 1.00 | 12.73 | 10.85 | 6.02 | 3.03 | 0.75 |
| **30** | Meziad cave | MZ 1 | 17.75 | 5.12 | -10.33 | 27.21 | 4.18 | 22.26 | 13.04 | 4.30 | 17.84 | 5.21 | 25.98 | 19.26 | 3.72 | 12.68 | 2.77 |
| **31** | Meziad cave | MZ 2 | 9.50 | 5.79 | -1.61 | 10.46 | 1.97 | 14.36 | 11.25 | 7.29 | 7.13 | 2.26 | 14.82 | 13.68 | 4.75 | 3.46 | 0.95 |
| **32** | Meziad cave | MZ 3 | 11.58 | 9.23 | 5.69 | 5.73 | 1.00 | 14.32 | 12.71 | 10.39 | 4.00 | 1.17 | 15.02 | 13.86 | 8.49 | 3.67 | 0.92 |
| **33** | Meziad cave | MZ 4 | 13.35 | 11.93 | 10.99 | 2.61 | 0.56 | 16.56 | 14.70 | 13.33 | 3.00 | 1.05 | 19.56 | 16.16 | 11.08 | 6.17 | 1.42 |
| **34** | Meziad cave | MZ 5 | 13.44 | 12.57 | 12.09 | 1.00 | 0.34 | 15.10 | 14.13 | 13.44 | 1.92 | 0.52 | 16.68 | 14.79 | 11.80 | 4.01 | 1.08 |
| **35** | Meziad cave | MZ 6 | 12.98 | 12.05 | 11.69 | 1.34 | 0.38 | 13.58 | 13.20 | 12.98 | 0.66 | 0.20 | 13.97 | 13.22 | 11.44 | 2.34 | 0.66 |
| **36** | Polovragi cave | PO 1 | 11.96 | 4.57 | -5.69 | 16.85 | 3.08 | 18.82 | 11.43 | 5.37 | 13.32 | 3.52 | 21.26 | 16.36 | 4.78 | 10.16 | 2.10 |
| **37** | Polovragi cave | PO 2 | 8.67 | 8.28 | 7.81 | 1.00 | 0.20 | 8.85 | 8.71 | 8.65 | 0.00 | 0.05 | 8.85 | 8.61 | 8.10 | 0.00 | 0.09 |
| **38** | Polovragi cave | PO 3 | 8.56 | 8.44 | 8.35 | 0.00 | 0.04 | 8.58 | 8.46 | 8.39 | 0.00 | 0.04 | 8.54 | 8.44 | 8.38 | 0.00 | 0.04 |
| **39** | Stogu cave | ST 1 | 8.38 | 5.05 | -0.68 | 8.00 | 1.87 | 13.44 | 9.90 | 5.87 | 8.00 | 1.67 | 15.61 | 12.69 | 5.20 | 5.00 | 0.96 |
| **40** | Stogu cave | ST 2 | 9.29 | 8.00 | 7.39 | 2.00 | 0.46 | 9.41 | 8.73 | 8.13 | 1.00 | 0.44 | 9.64 | 9.49 | 7.72 | 0.00 | 0.07 |
| **41** | Stogu cave | ST 3 | 9.06 | 7.80 | 6.92 | 2.76 | 0.48 | 9.66 | 8.81 | 7.97 | 1.76 | 0.50 | 10.01 | 9.72 | 7.55 | 0.56 | 0.12 |
| **42** | Valea Cetăţii cave | VC 1 | 10.58 | 1.19 | -14.79 | 24.49 | 4.32 | 17.63 | 9.97 | 3.55 | 13.85 | 3.67 | 17.91 | 14.69 | 2.10 | 8.49 | 1.73 |
| **43** | Valea Cetăţii cave | VC 2 | 7.07 | 2.50 | -3.96 | 10.14 | 2.16 | 9.47 | 7.81 | 5.31 | 4.13 | 1.08 | 9.53 | 8.69 | 3.04 | 2.57 | 0.49 |
| **44** | Valea Cetăţii cave | VC 3 | 8.17 | 6.25 | 2.68 | 6.00 | 1.06 | 8.79 | 8.38 | 7.60 | 1.00 | 0.28 | 8.81 | 8.26 | 5.98 | 1.00 | 0.33 |
| **45** | Valea Cetăţii cave | VC 4 | 8.06 | 6.07 | 2.64 | 5.93 | 1.07 | 8.67 | 8.31 | 7.67 | 1.00 | 0.25 | 8.68 | 8.13 | 6.25 | 1.00 | 0.36 |
| **46** | Valea Cetăţii cave | VC 5 | 8.16 | 6.41 | 3.32 | 5.00 | 0.97 | 8.78 | 8.39 | 7.68 | 1.00 | 0.26 | 8.80 | 8.25 | 6.01 | 1.00 | 0.32 |
| **47** | Valea Cetăţii cave | VC 6 | 8.22 | 6.90 | 4.71 | 3.99 | 0.71 | 8.59 | 8.31 | 7.97 | 1.00 | 0.17 | 8.53 | 8.01 | 6.52 | 1.00 | 0.31 |

Table S2 – Errors for the interpolated environmental datasets generated with the natural neighbour method via-cross validation: dependent dataset (D – data loggers), the independent dataset (S – spot measurements).

| **No.** | **Cave** | **No. obs. D** | **MAE D** | **RMSE D** | **No. obs. S** | **MAE S** | **RMSE S** |
| --- | --- | --- | --- | --- | --- | --- | --- |
| 1 | Gura Dobrogei | 4392 | -0.3 | 0.9 | 270 | -0.4 | 0.9 |
| 2 | Canara | 4392 | -0.3 | 0.3 | 126 | -0.2 | 0.4 |
| 3 | Hagieni | 4392 | 0.2 | 0.3 | 162 | 0.3 | 0.4 |
| 4 | Valea Cetăţii | 8748 | 0.1 | 0.6 | 162 | -0.2 | 0.7 |
| 5 | Muierilor | 11664 | 0.2 | 0.2 | 234 | 0.1 | 0.4 |
| 6 | Polovragi | 4374 | 0.1 | 0.1 | 126 | 0.2 | 0.3 |
| 7 | Stogu | 4381 | -0.1 | 0.4 | 180 | -0.3 | 0.5 |
| 8 | Lacul Verde | 4381 | -0.3 | 0.4 | 180 | -0.2 | 0.4 |
| 9 | Closani | 13122 | 0.1 | 0.4 | 252 | 0.2 | 0.5 |
| 10 | Meziad Level 1 | 4374 | -0.2 | 0.3 | 144 | -0.3 | 0.5 |
| 11 | Meziad Level 2 | 5832 | 0.2 | 0.4 | 216 | 0.1 | 0.2 |

Table S3 – Spot data summary statistics per activity period for the observed species within the SEs. D - Distance from the observed animals to the nearest SE entrance, H – height of the observation relative to the SE floor, T – spot temperature measured near the animal.

| **No** | **Species** | **Activity period** | **Avg_D (m)** | **Max_D (m)** | **Min_D (m)** | **Avg_H (m)** | **Max_H (m)** | **Max_H (m)** | **Avg_T (°C)** | **Max_T (°C)** | **Min_T (°C)** | **StDev_T** | **Range_T(°C)** |
| --- | --- | --- | --- | --- | --- | --- | --- | --- | --- | --- | --- | --- | --- |
| 1 | *Miniopterus schreibersii* | AM | 44.83 | 111.41 | 30.8 | 4.84 | 8.2 | 1.7 | 10.79 | 12.06 | 3.57 | 1.18 | 8.49 |
|  |  | NM | 117.26 | 650.29 | 6.3 | 6.03 | 21.5 | 0.6 | 7.42 | 13.97 | 0.21 | 1.78 | 13.76 |
|  |  | SO | 53.39 | 73.41 | 28.62 | 6.89 | 8.3 | 3.1 | 9.98 | 10.06 | 8.43 | 0.11 | 1.63 |
| 2 | *Myotis myotis/blythii* | AM | 49.79 | 164.52 | 23.67 | 4.96 | 14.5 | 2.3 | 11.2 | 12.41 | 9.74 | 0.36 | 2.67 |
|  |  | NM | 122.63 | 286.73 | 2.2 | 10.21 | 15.2 | 1.4 | 7.5 | 12.31 | 5.6 | 1.74 | 6.71 |
|  |  | SO | 106.04 | 662.94 | 12.2 | 3.12 | 8 | 0.9 | 12.06 | 13.2 | 8.43 | 1.91 | 4.77 |
| 3 | *Nyctalus noctula* | NM | 107.64 | 109.72 | 53.45 | 15.02 | 15.2 | 7.3 | 6.36 | 8.41 | 6.3 | 0.32 | 2.11 |
| 4 | *Pipistrellus pipistrellus* | NM | 60.71 | 142.47 | 39.61 | 6.67 | 15.2 | 1 | 8.35 | 8.45 | 6.3 | 0.2 | 2.15 |
| 5 | *Rhinolophus euryale* | AM | 174.58 | 511.19 | 1.98 | 5.08 | 14.5 | 1.4 | 11.49 | 12.77 | 8.07 | 1.41 | 3.42 |
| 6 | *Rhinolophus ferrumequinum* | AM | 142.68 | 220.31 | 43 | 7.05 | 14.5 | 1.7 | 11.73 | 12.97 | 9.74 | 0.97 | 3.23 |
|  |  | NM | 78.4 | 286.74 | 2.2 | 4.95 | 15.2 | 0.75 | 8.14 | 12.92 | 3.84 | 1.2 | 9.08 |
|  |  | SO | 134.27 | 677.59 | 6.93 | 4.48 | 14.6 | 0.6 | 9.91 | 14.04 | 7.37 | 0.89 | 6.67 |
| 7 | *Rhinolophus hipposideros* | AM | 130.78 | 164.44 | 122 | 12.96 | 14.5 | 6.8 | 10.29 | 12.4 | 9.74 | 1.11 | 2.66 |
|  |  | NM | 71.72 | 130.71 | 2.2 | 7.36 | 15.2 | 1.4 | 7.91 | 11.93 | 5.6 | 1.49 | 6.33 |
|  |  | SO | 80.61 | 150.3 | 46.28 | 4.3 | 8 | 1.3 | 9.5 | 10.16 | 8.43 | 0.79 | 1.74 |
| 8 | *Rhinolophus mehelyi* | NM | 69.84 | 286.7 | 6.5 | 3.64 | 7 | 2 | 8.4 | 12.31 | 6.81 | 2.02 | 5.5 |
