# Supplementary material for: Bat dynamics modelling as a tool for conservation management in subterranean environments
Source: PLoS One. 2022 Oct 20;17(10):e0275984. doi: 10.1371/journal.pone.0275984 (PMC9584375; doi:10.1371/journal.pone.0275984)
Supplement: Attachment — Submitted filename: PLOS_bat dynamics_2022.pdf [file pone.0275984.s002.pdf]

I greatly appreciate the opportunity to have read your manuscript entitled “Bat dynamics modelling as a tool for conservation management in show caves”. I quite found the research itself very exciting due to the specific recommendations for management actions, and these recommendations were derived using information acquired by ensemble SDMs. Ensemble SDMs are a technique that I have not read about, so I was happy to learn a new modelling method. I think that the research is very fascinating and is a great addition to the literature. However, I feel that there are portions of the paper that might require a bit of restructuring and editing. I have written my recommendations below. Hopefully they are useful to you.

Title: What I find interesting is that the title seems geared toward just show caves, but some of the sites you monitored were mines and other non-show caves. You might want to consider adjusting the title to make it less restrictive. The methods used can be applied for both non-tourist and tourist sites, and that’s neat!

Abstract: I think that the abstract clearly stated what the research was about, had clear justification and a nice summary of results. Very succinct. I have a couple minor edits:

Line 48: I would say (e.g., caves and mines) as bats are in other locations too. I do know that your paper is just about caves and mines, but I think it’s fine for the abstract to add in the “e.g.”
Line 50: You can remove “may” and just say “if optimal patches become unavailable”

Introduction: The introduction was well-written. I think it provided good background information to introduce the topic. However, I think perhaps you could briefly mention a little bit about ensemble SDMs. I noted that it was said why there are pitfalls to SDM modelling of SEs, but then ensemble SDMs are just mentioned as the method. Perhaps you could split up line 143 and 144, so that in the second sentence you can say something very brief about it (e.g., the benefit of this model type). The Woodman et al. 2019 manuscript that describes the ensemble SDM could be good here perhaps.

Here, and throughout, check that you use the hypen for “cave-dwelling” and “crevice-dwelling” to stay consistent.

Line 75: should it be “and” instead of “or” when you mention RELCOM? E.G: “and RELCOM 2007”
Line 76: “cave-dwelling bat species” – remove the ‘s’ on bats
Line 86: just nitpicky, but “select” could be used instead of “choose”
Line 118: missing a space between “et al. 1998”
Line 142: remove a period
Line 149: Something here is amiss. Perhaps it should say “This novel approach was challenged by a series of limiting factors…”

Methods: I keep wondering if already in the methods the species should be mentioned as the analyses and results are species-specific. I think this is my largest suggestion for the methods section. I only mention this (and probably state it below a few times) as I was made aware of these species in the results and it was unexpected.

Check the use of hyphens versus en-dash throughout. The en-dash is used for ranges (e.g. September–October; e.g. line 166).

I might recommend putting more information into the table descriptions so that they better "stand alone" unless the journal says otherwise.

Line 156: "We chose" instead of "have chosen"
Line 160/161: I would say in one sentence that you used flash photography. I would then state in another sentence that the work was permitted by the Romanian Academy. Two different topics.
Line 162: I think this should come before the sentence on permitting.
Line 169: I think that the years sampled could potentially fit into Table 1. Then you can just say "…and each SE was observed for two consecutive years between 2010 and 2016 (Table 1)"
Line 170: What do you mean by "later period"?
Line 171: I am not sure what you mean when you say "It was considered relevant". Or perhaps, what is the purpose of this sentence? Are you trying to say that surveys were useful for model development?
Line 173: I might say "The spatial extend of SEs was…"
Line 174: Suggested edit: "The spatial extent of SE 1–3 was performed…"
Line 178: What is the purpose of merging the maps in a single layout if the maps have different spatial references?
Line 179: "mapping bat occurrences"? Might add "bat" for clarity
Line 187: I would rewrite and say "We used the number of passes recorded by people counters (tourist passes) as a proxy for the number of tourists who visited the caves.
Line 189: change "since" to "because". Since refers to time
Line 190: you say you considered attributing 100 passes, but you did do this. I would say "We attributed 100 human passes per year for scalability"
Line 199: the degree symbol is before the C. should be flipped. Also the C is missing from the other value
Line 218: change "holds" to "held"
Line 222: Suggested edit "We included data on human-induced temperature changes (resulting from tourist presence and light systems) collected from show caves used in our study in our approach…". Then you can get rid of the sentence on line 224.
Line 224–233: All the information about temperatures read to me as results. I recommend moving.
Line 244: I use a citation for the > 10 for VIF (e.g., Bowerman, B. L., and R. T. O'Connell. 1990. Linear statistical models: an applied approach. 2nd ed. PWS Kent Publishers, Belmont, California, Duxbury.)
Line 265: "coefficient, SSDM R package" just remove the hyphen and use a comma

Line 268: remove “have”, e.g., “we used a ten-percentile..”
Line 273: This is a result, and I would reference table 2 with this.
Line 273/274: This makes me wonder if the species should already be mentioned in the methods given how the models were developed.
Line 275: Move to results section. I would also then take the last two sentences of that paragraph and put into the previous paragraph (perhaps just add to the end of the previous paragraph).

Results: I think this section needs a bit of work so that it’s a bit easier to follow. I believe there are some methods mixed into the Results section that hinder the clarity. I’ve tried to identify where this happens. I also found that perhaps some of the analyses were not stated earlier, or at least were not clear as I could not match them to the results. Check for consistency on how you write the “p” for “P-value” as sometimes it’s capitalized and other times its lower case and italicized.

Line 282: This sentence is a method if you are saying that they were considered a single group for modelling purposes. If that is the case, then the species might need to be identified in the methods section.
Line 286: change “has been” to “was”
Line 288: I think the part about selecting high temperature range variation is fine, but mentioning “to raise young” seems like a point for discussion and not really a result. I might just remove this part.
Line 294–297: This reads as methods to me.
Line 297: What do you mean by “acceptable values”? I would elaborate on this sentence in general.
Line 303: This again reads as a method. The next sentence is the result of the method.
Line 306: The friedman’s test was only mentioned for the number of species in each SE. Might need to check the statistical analysis section if you did an analysis on number of bats. Also, was this summed across all SEs or for individual SEs? I would clarify that in the analysis section too.
Line 320: This is a method.
Line 332/335: I would just list the species rather than saying “most”. Or say, “all species except XX”
Line 358: So, I think I need the justification as to why the models were summarized this way. Perhaps this relates to the statements in the discussion (see line 429)?

Table 3, 4: Check how many decimal places you use for the P-values. I might recommend also matching to the results section to be consistent.

Figures 3–5: I think it would be beneficial to add to the description what the values mean, especially for someone who is unfamiliar with this type of modelling.

Discussion: This section I had the most difficulties with. The analyses, as I followed, were very species-specific, and yet I am lacking a discussion on these species-specific results. Even a

discussion on the clustering of species within the sites could be better discussed. I am going to assume that the recommendations for the management of each SE are derived in part from these species, and so I would recommend discussing what you found for the bat species. Further, there are some results in the discussion that should be moved. I would also recommend not using the acronyms/letters in the discussion. I would just say “early hibernation” for instance. It makes it a bit easier to read. I think those are fine to use in the methods and results.

Line 384 and 386: I think references are needed.

Paragraph starting on line 387: This all reads as results to me. You are mentioning your findings. In the discussion you should say how these findings broadly relate to the literature. You have species-specific info here: do your results match other studies? I think the last two sentences are discussion points.

Line 401: I would expand. Break the sentence apart and reference some literature on the benefits of warm areas for bats during maternity. Could compare to how low temperatures are perhaps not ideal for rearing pups.

Line 405, and most of this paragraph, read as methods. It’s the justification for why you ran the models you did. This section is listed as “Species distribution models” and yet there is no discussion on the outcomes of the models themselves in relation to the current literature.

Is the paragraph starting with line 422 about the pros/cons to SDM versus the ensemble modelling?

Line 429–432: Methods and results. Please move to appropriate areas.

Paragraph starting with line 433: I think there are good things here, but that some more relation to the literature is needed. For example, you mention that the minimum temperature is important at the beginning of hibernation. Is this result the same seen in the literature?

Line 441: Is the statement about optimal torpor temps your finding or something from literature? If literature, it needs a citation.

Paragraph starting line 447 is results. Discuss more, for example, why you think the SDM values are higher in more complex systems.

Line 452: What do you mean by “sensible species”?
Line 453–455: methods; also I think you mean “susceptible to human disturbance” and not “sensible” here

Line 470: this seems to be a description of a site (method) rather than a discussion point

Lines 473–493: I am just not really seeing a discussion with the literature. Some of this is a description of the site which would go in the methods.
