# Supplementary material for: Bat dynamics modelling as a tool for conservation management in subterranean environments
Source: PLoS One. 2022 Oct 20;17(10):e0275984. doi: 10.1371/journal.pone.0275984 (PMC9584375; doi:10.1371/journal.pone.0275984)
Supplement: Attachment — Submitted filename: Response to reviewers.docx [file pone.0275984.s004.docx]

**Dear reviewers,**

We have addressed all the typos and errors mentioned in this new review, with one specific mention:

**Reviewer#1**
Line 318: should it be 250.000 or 250,000?

Response: We eliminated the coma or point within this number to avoid any misunderstandings; it was a quarter of a million.

We would like to express our sincere gratitude for the kind words and for your intense work for this manuscript. The changes you have suggested helped us greatly improve the manuscript, with constructive and concise arguments. We feel that this was one of the most helpful reviews we processed.

We have included your contributions to our acknowledgements.

**Dear editors,**

We would like to express our sincere gratitude for your intense work for this manuscript.

We have corrected the reviewer suggestions and we are attaching the clean manuscript plus the track changes version. We have also corrected the references and double checked them.

We also added this line to the acknowledgments section: “We would like to thank the editors and reviewers for their efforts, acknowledging that their suggestions greatly enhanced this manuscript”.

Sincerely yours,

The authors
